# The impact of COVID-19 restrictions on Australians' frequency and duration of participation in different types of sport and physical activity

**DOI:** 10.1186/s13102-022-00435-z

**Published:** 2022-03-21

**Authors:** Rochelle Eime, Jack Harvey, Melanie Charity, Aurelie Pankowiak, Hans Westerbeek

**Affiliations:** 1 School of Science, Psychology and Sport, Federation University, Ballarat, Australia; 2 Institute for Health and Sport, Victoria University, Melbourne, Australia

**Keywords:** Sport, Participation, Frequency, Duration, COVID-19

## Abstract

**Background:**

Sports management and public health physical activity stakeholders need to understand changing patterns of participation to inform the development of sport and physical activity opportunities and strategies. This study investigated changes in the frequency and duration of participation in sport and physical activity in Australia from pre-COVID-19 to during-COVID-19, broken down by the specific type of activity and by gender, age and region.

**Methods:**

During the first pandemic restrictions and lockdowns in Australia in May–June 2020, 6140 survey respondents provided information about the types, frequency and duration of the sport and physical activity they participated in prior to and during COVID-19 restrictions. Differences between mean values were analyzed.

**Results:**

The greatest decline in participation during COVID-19 was in team sports, and the decline was greater for men than for women.

**Conclusion:**

How will sport respond to getting these men back in the game, and women back from home-based yoga and Pilates?

## Background

All over the world the COVID-19 restrictions have limited people’s opportunities to be active. With organized and competitive participation in sport cancelled, mainly younger people, were either forced to take up another form of sport or activity, participate in online organized or non-organized activities, or stop participating altogether [1]. During various stages of the COVID-19 pandemic, many countries have closed schools, which has limited children’s’ physical education and free-play. During the latest lockdown in Victoria, Australia, even playgrounds for young children were closed, leaving preschool children with very limited opportunities to be active [2].

For sedentary individuals, COVID-19 restrictions would likely have made it more difficult to initiate activity, however for some it may have been a catalyst to start a more active life. If individuals can be physically active, and continue to be active, even when they are restricted in their ability to leave their own home, there will be clear and obvious physical and mental health benefits resulting from maintaining an active lifestyle [3]. However, staying at home can lead to a lot of stress, anxiety and mental distress. Some authors argue that the best way to overcome these problems is to replace outdoor activities like sport, with home-based activities, such as bodyweight training and dance-based aerobic exercise, and if possible, aerobic high-intensity exercise using stationary bikes or rowing ergometers, with self-paced protocols [3].

Before the COVID 19 pandemic, there were some consistent global patterns of participation in physical activity and sport. Participation in sport was most prevalent among children and youth, whereas adults were more likely to be active through other leisure-time pursuits such as walking, cycling and running [4]. Children and youngsters aged 5–14 years were most engaged, with significant declines in participation occurring from age 15 years [5]. During adolescence both participation in sport and total physical activity levels decline [6].

Further, prior to the pandemic there were consistent trends of young people gradually transferring their participation away from organized club and/or team-based sports activities to non-organized sport or general leisure-time physical activity [1, 7, 8]. These non-organized activities could include informal pick-up participation like park basketball or spontaneous play [1]. There was also a shift away from traditional club-based sports like baseball or hockey to newer sports such as rock climbing or skateboarding [1]. A recent Australian study reported the majority of sport participation for children was through a local sports club, whereas adults were less likely to participate in sport through a sports club, and often engaged in sport-like physical activities through gyms and other community recreational settings [8].

Whilst there were emerging societal changes in regard to participation in sport and physical activity pre-COVID-19, the pandemic has had an unprecedented global impact on participation in sport and physical activity [9–11].

A scoping review of 150 studies across 26 countries regarding children and youth physical activity behaviors during COVID-19 found that studies consistently reported declines in physical activity time, as well as increases in screen time and sedentary behaviors [11]. Cross-sectional and longitudinal studies consistently reported decreases in physical activity time for children and youth; however, some reported decreases in participation in sport but not in non-organized physical activities [11]. The general consensus between studies was a shift from outdoor and community activities to home-based activities [11]. This is similar to other systematic reviews that investigated physical activity during COVID-19. These also concluded that the majority of studies reported a decrease in physical activity and increases in sedentary behavior across all population groups [12, 13].

A German study reported that many of those who were active through leisure-time sport or exercise pre-COVID-19, completely stopped being active or reduced activity by at least 1 h per week. However, nearly a third maintained their usual levels of activity, and a few increased activity [10]. For those who reduced or stopped their activity during lockdown, many mentioned that this was due to the closing of sports facilities [10]. Another study of sport-related activity reported that whilst sport was cancelled during COVID-19, many sports organizations utilized media technologies such as social media platforms to stay engaged with their participants or players [9].

COVID-19 has impacted individuals’ sport and physical activity participation in different ways, in regard to activity status pre-COVID but also in regard to a range of personal characteristics and experiences during COVID-19, with some demographic groups impacted more severely than others. For example, a study of working adults reported that leisure-time physical activity was more negatively impacted for working mothers than fathers, due to a greater burden on mothers who had to juggle work demands and childcare duties including home-schooling [14]. In addition, given the differences in participation in sport and physical activity patterns across age groups pre-COVID-19, the pandemic is likely to have exacerbated these difference in participation patterns across age groups. Further, participation in sport and physical activity also differs across metropolitan and non-metropolitan areas. Participation in sport in non-metropolitan areas is underpinned by a strong culture of community sport, and there is less choice of leisure-time activities [15], which may lead to regional differences in the effect of COVID-19 on participation.

To date, most studies on the effects of COVID-19 on physical activity have reported overall activity levels, and some have reported the type of activity in general (e.g., sport and non-sport), but none report frequency and duration across specific types of activity. Therefore, the aim of this study is to investigate changes in the frequency and duration of participation in sport and physical activity in Australia from pre-COVID-19 to during-COVID-19, broken down by the specific type of activity and by gender, age and region.

## Methods

This study is part of a broader program of research in Australia which involves the longitudinal measurement of sport and physical activity profiles and physical, mental and social health and wellbeing outcomes that are the result of this participation.

The present study is based on data collected in the first wave of data collection which included retrospective (baseline) data pertaining to pre-COVID-19 (2019) as well as during COVID-19 restrictions (2020). An online survey of sport participants was conducted during May and June 2020 using the Qualtrics survey tool. Recruitment to the survey was primarily facilitated by national and state sporting organizations. The target population was people aged 13 years or older who were registered in the 2019 and/or 2020 playing seasons to participate in one or more sports. The sport organizations that sent out the survey invitation to their registered participants represent major sports in Victoria and Australia [8, 16]. The research team has extensive research experience in working with these sports at national, state and local levels [15, 17–21].

In order to broaden the scope of the survey sample to include people who participate in recreational physical activity only, in settings other than sports clubs, the primary recruitment strategy was supplemented by the use of snowball sampling, through social media pages of sport organizations and research-oriented social media pages of the research team.

The first wave, or baseline, of the longitudinal survey included among other themes, questions about:

- Demographic characteristics—gender, age, and residential postcode
- Types of sports and other recreational physical activities participated in
- Frequency and duration of participation, at the time of the survey (May–June 2020) and during the previous year (2019).

Date of birth was used to determine age in years at the time the survey was completed. Age was then recoded into two age cohorts: adolescents (13–17 years) and adults (18 years and above). Residential postcode correspondence tables [22] were used to assign each postcode to one of two broad geographical zones or regions: Metropolitan, comprising the capital cities of the Australian states; and Non-metropolitan, comprising regional cities, towns and rural areas.

For each included sport and physical activity, the mean values for frequency and for duration were tabulated by gender and region. Differences between mean values were analyzed by repeated measures analysis of variance (RMANOVA) with three effects (time, group, time × group interaction). Statistical significance was set at *p* < .05. Analyses were conducted using SPSS version 24.

Three sets of two tables were prepared, showing breakdowns by gender and region for each of: all respondents; adults; and adolescents. Tables 1 and 2 (all respondents) are included in the body of the paper. Tables 3, 4, 5 and 6 (adults and adolescents) are supplementary tables. Because of differences in the profiles of sports and physical activities undertaken by adults and adolescents, there are corresponding differences in the activities selected for inclusion individually in each set of tables. More details are provided in the results section.

**Table 1:** Changes in self-reported frequency and duration of 18 popular forms of sport and physical activity^1^ from before Covid to during Covid: persons by gender

Activity	Gender^2^	n^2,3^	Frequency^4^ (Sessions per two weeks)			Duration^5^ (Minutes per session)		
*p* values^6^			Mean before Covid	Mean during Covid	Change %	Mean before Covid	Mean during Covid	Change %
Athletics	Male	98	2.95	1.98	− 32.9	46.79	22.62	− 51.7
	Female	63	5.41	3.47	− 36.0	58.02	33.90	− 41.6
	All	162	3.90	2.54	− 35.0	51.05	26.87	− 47.4
*p *values			Year .019 Gender .032			Year < .001 Gender .049		
Australian football	Male	472	2.75	1.03	− 62.4	66.79	24.32	− 63.6
	Female	131	2.58	0.60	− 76.6	65.17	14.60	− 77.6
	All	604	2.72	0.94	− 65.4	66.45	22.14	− 66.7
*p *values			Year < .001			Year < .001		
Basketball	Male	224	2.98	2.16	− 27.5	46.76	22.22	− 52.5
	Female	113	2.29	1.57	− 31.4	44.06	10.92	− 75.2
	All	337	2.75	1.96	− 28.6	45.86	18.51	− 59.6
*p *values			Year .016			Year < .001 Gender .019		
Bowls	Male	807	6.23	0.53	− 91.5	152.25	36.85	− 75.8
	Female	290	5.90	0.33	− 94.4	171.34	36.72	− 78.6
	All	1104	6.19	0.47	− 92.3	157.32	36.74	− 76.6
*p *values			Year < .001			Year < .001 Year × Gender .017		
Boxing	Male	157	2.30	1.21	− 47.4	38.56	14.11	− 63.4
	Female	50	1.69	3.02	78.7	31.97	17.81	− 44.3
	All	208	2.14	1.63	− 24.0	36.80	14.87	− 59.6
*p *values			Year × Gender .042			Year < .001		
Bush walking	Male	253	1.74	2.17	24.2	90.51	43.73	− 51.7
	Female	252	1.80	2.09	16.0	93.39	46.51	− 50.2
	All	507	1.77	2.12	20.0	92.01	45.07	− 51.0
*p *values			Year < .001					
Cricket	Male	509	3.17	0.59	− 81.3	128.62	32.30	− 74.9
	Female	65	2.51	0.60	− 75.9	79.16	11.31	− 85.7
	All	575	3.09	0.59	− 80.8	122.81	29.82	− 75.7
*p *values			Year < .001			Year < .001 Gender < .001		
Cycling	Male	709	3.52	4.10	16.7	60.94	57.47	− 5.7
	Female	388	2.73	3.48	27.2	47.00	50.39	7.2
	All	1102	3.24	3.89	19.9	55.88	54.89	− 1.8
*p *values			Year .002 Gender .003			Gender .002		
Fitness/gym	Male	837	4.92	3.39	− 31.2	55.03	28.97	− 47.4
	Female	717	4.19	3.58	− 14.7	54.12	27.55	− 49.1
	All	1557	4.59	3.48	− 24.2	54.58	28.30	− 48.2
*p *values			Year < .001 Year × Gender .002			Year < .001		
Football/soccer	Male	986	5.52	2.52	− 54.4	89.88	33.64	− 62.6
	Female	379	4.53	1.80	− 60.2	86.06	28.03	− 67.4
	All	1372	5.25	2.31	− 56.0	89.08	32.07	− 64.0
*p *values			Year < .001 Gender .001			Year < .001 Gender .031		
Golf	Male	1556	4.28	1.47	− 65.6	218.18	58.14	− 73.4
	Female	686	4.57	1.17	− 74.3	229.06	50.64	− 77.9
	All	2247	4.37	1.38	− 68.5	221.54	55.83	− 74.8
*p *values			Year < .001			Year < .001 Year × Gender .002		
Netball	Male	25	2.36	0.68	− 71.2	63.08	12.17	− 80.7
	Female	162	3.04	0.68	− 77.5	57.00	13.50	− 76.3
	All	189	2.96	0.68	− 77.1	58.27	13.16	− 77.4
*p *values			Year .013			Year < .001		
Pilates	Male	66	2.55	1.44	− 43.3	52.73	27.50	− 47.8
	Female	236	2.24	1.94	− 13.5	46.86	24.33	− 48.1
	All	302	2.31	1.83	− 20.6	48.12	25.04	− 48.0
*p *values			Year < .001 Year × Gender .017			Year < .001		
Running/jogging	Male	642	3.64	4.71	29.7	34.92	32.36	− 7.3
	Female	414	3.14	4.63	47.5	33.45	33.71	0.8
	All	1060	3.44	4.68	36.0		32.95	− 4.2
*p *values			Year < .001					
Swimming	Male	528	4.45	0.78	− 82.5	58.58	13.24	− 77.4
	Female	576	6.05	1.33	− 77.9	77.64	13.77	− 82.3
	All	1110	5.28	1.07	− 79.8	68.53	13.48	− 80.3
*p *values			Year < .001 Gender < .001 Year x Gender .005			Year < .001 Gender < .001 Year x Gender < .001		
Tennis	Male	488	3.45	1.44	− 58.2	97.69	39.20	− 59.9
	Female	370	3.26	1.19	− 63.6	110.76	33.66	− 69.6
	All	861	3.37	1.33	− 60.5	103.29	37.01	− 64.2
*p *values			Year < .001			Year < .001 Year x Gender .001		
Walking	Male	1405	7.00	8.60	22.8	53.20	50.76	− 4.6
	Female	1159	6.52	8.61	32.1	58.16	55.47	− 4.6
	All	2571	6.79	8.61	26.8	55.42	52.90	− 4.5
*p *values			Year < .001					
Yoga	Male	74	3.09	3.23	4.4	34.47	21.34	− 38.1
	Female	230	2.60	3.32	27.6	44.30	27.95	− 36.9
	All	308	2.72	3.30	21.2	41.69	26.31	− 36.9
*p *values			Year < .001 Gender .001					
Other activities ^6,7^	Male	573	2.61	1.25	− 52.1	84.99	31.41	− 63.0
	Female	584	3.13	2.10	− 33.0	77.57	28.36	− 63.4
	All	1166	2.91	1.68	− 42.4	81.23	29.68	− 63.5
All activities^7^	Male	10,409	4.48	2.93	− 34.5	97.42	39.26	− 59.7
	Female	6865	4.26	3.26	− 23.5	85.57	35.63	− 58.4
	Other^2^	31	5.35	2.10	− 60.8	67.90	16.83	− 75.2
	No response^2^	37	6.57	2.94	− 55.2	105.81	32.70	− 69.1
	All	17,342	4.40	3.06	− 30.4	92.69	37.78	− 59.2

^1^Of the 6140 survey respondents 5371 answered the questions on sport and physical activities. They reported 19,205 instances of participation (i.e. a person reporting participation in a particular activity) in a total of 88 sports and physical activities—an average of 3.6 different activities per person. The 18 activities which each contributed at least 1% of all instances of participation are listed in this table

^2^Four gender response categories were provided: ‘Male’, ‘Female’, ‘Other’, and ‘Choose not to respond’ (abbreviated to ‘No response’ in the table). The ‘Other’ and ‘No response’ categories had counts, across all activities, of 31 and 37 respectively. The maximum count for an individual activity was 6. With such small sample sizes, the evidence base is small and the sampling variability is extremely large, and so for these categories the results for individual activities are not included in the table. For the aggregate results of all activities combined, results for all four gender categories are included

^3^Some respondents did not answer the frequency and duration questions, and so the total number of responses is 17,342 rather than 19,205. A few gave partial responses, and so the sample sizes for the four variables in this table are not exactly equal. The indicative sample sizes shown in the table are for the frequency before Covid

^4^Question wording for frequency: “Before the Coronavirus (COVID-19) pandemic, in a usual 2-week period during the season for each of these sports, how many times would you participate (including any training or practice)?”; “In the past two weeks, how many times have you participated in each of these sports (including any training or practice)?”

^5^Question wording for duration: “On average, how many minutes would each session last?”; “On average, how many minutes does each session last?”

^6^Only statistically significant *p *values (< .05) are shown in the table

^7^ ‘Other’ encompasses the 70 activities which each contributed less than 1% of all reported instances of participation

^8^No statistical significance tests were conducted for data aggregated across multiple activities because many respondents participated in more than one activity, violating the assumption of independent observations

**Table 2:** Changes in self-reported frequency and duration of 18 popular forms of sport and physical activity^1^ from before Covid to during Covid: persons by region

Activity	Region^2^	n^3^	Frequency^4^ (sessions per 14 days)			Duration^5^ (minutes per session)		
*p *values^6^			Mean before Covid	Mean during Covid	Change %	Mean before Covid	Mean during Covid	Change %
Athletics	Metro	116	3.18	2.48	− 22.1	50.65	29.42	− 41.9
	Non-metro	45	5.76	2.64	− 54.1	51.85	19.77	− 61.9
	All	161	3.90	2.53	− 35.3	50.99	26.65	− 47.7
*p *values			Year .006			Year < .001		
Australian football	Metro	366	2.57	0.95	− 63.0	66.32	23.64	− 64.3
	Non-metro	237	2.96	0.92	− 68.9	66.94	19.94	− 70.2
	All	603	2.72	0.94	− 65.5	66.56	22.18	− 66.7
*p *values			Year < .001			Year < .001		
Basketball	Metro	241	2.80	2.25	− 19.7	44.59	20.18	− 54.7
	Non-metro	94	2.65	1.28	− 51.6	49.41	14.47	− 70.7
	All	335	2.76	1.97	− 28.7	45.95	18.63	− 59.5
*p *values			Year .003			Year < .001 Region .020 Year x Region .020		
Bowls	Metro	713	6.48	0.50	− 92.2	160.86	35.72	− 77.8
	Non-metro	388	5.66	0.42	− 92.6	151.23	38.98	− 74.2
	All	1101	6.19	0.47	− 92.4	157.50	36.85	− 76.6
*p *values			Year < .001			Year < .001		
Boxing	Metro	138	1.89	0.99	− 47.4	38.30	14.11	− 63.2
	Non-metro	68	2.64	2.91	10.3	33.06	15.50	− 53.1
	All	206	2.14	1.63	− 23.9	36.58	14.55	− 60.2
*p *values			Region .049			Year < .001		
Bush walking	Metro	295	1.70	1.91	12.3	94.97	45.17	− 52.4
	Non-metro	212	1.85	2.40	29.6	87.96	44.93	− 48.9
	All	507	1.77	2.12	20.0	92.01	45.07	− 51.0
*p *values			Year < .001					
Cricket	Metro	393	3.04	0.54	− 82.1	124.45	31.62	− 74.6
	Non-metro	179	3.18	0.69	− 78.4	119.22	26.39	− 77.9
	All	572	3.09	0.59	− 80.9	122.83	29.99	− 75.6
*p *values			Year < .001			Year < .001		
Cycling	Metro	703	3.32	3.89	17.3	59.24	57.96	− 2.2
	Non-metro	397	3.12	3.87	24.2	50.12	49.58	− 1.1
	All	1100	3.25	3.89	19.7	55.94	54.93	− 1.8
*p *values			Year < .001			Region .012		
Fitness/gym	Metro	1094	4.57	3.53	− 22.7	54.93	29.29	− 46.7
	Non-metro	457	4.67	3.33	− 28.6	53.99	26.12	− 51.6
	All	1551	4.60	3.47	− 24.5	54.65	28.36	− 48.1
*p *values			Year < .001			Year < .001		
Football/soccer	Metro	1055	5.33	2.36	− 55.7	87.62	33.39	− 61.9
	Non-metro	313	4.98	2.15	− 56.9	94.13	27.93	− 70.3
	All	1368	5.25	2.31	− 56.0	89.11	32.14	− 63.9
*p *values			Year < .001			Year < .001 Year x Region .001		
Golf	Metro	1256	4.17	1.37	− 67.1	225.35	54.49	− 75.8
	Non-metro	987	4.64	1.39	− 70.1	216.82	57.59	− 73.4
	All	2243	4.38	1.38	− 68.5	221.60	55.85	− 74.8
*p *values			Year < .001			Year < .001		
Netball	Metro	102	3.29	0.70	− 78.9	59.56	11.79	− 80.2
	Non-metro	87	2.56	0.65	− 74.5	56.73	14.88	− 73.8
	All	189	2.96	0.68	− 77.1	58.27	13.16	− 77.4
*p *values			Year < .001			Year < .001		
Pilates	Metro	209	2.34	1.80	− 23.0	49.95	26.22	− 47.5
	Non-metro	93	2.23	1.90	− 14.7	44.02	22.23	− 49.5
	All	302	2.31	1.83	− 20.6	48.12	25.04	− 48.0
*p *values			Year .033			Year < .001 Region .044		
Running/jogging	Metro	745	3.29	4.69	42.8	34.56	32.95	− 4.7
	Non-metro	312	3.84	4.66	21.4	34.11	33.03	− 3.2
	All	1057	3.45	4.68	35.8	34.43	32.98	− 4.2
*p *values			Year < .001					
Swimming	Metro	732	5.22	1.04	− 80.0	69.50	13.81	− 80.1
	Non-metro	373	5.44	1.13	− 79.2	66.85	12.92	− 80.7
	All	1105	5.29	1.07	− 79.7	68.61	13.51	− 80.3
*p *values			Year < .001			Year < .001		
Tennis	Metro	610	3.51	1.24	− 64.7	104.78	38.41	− 63.3
	Non-metro	250	3.02	1.55	− 48.5	99.63	33.66	− 66.2
	All	860	3.37	1.33	− 60.5	103.27	37.06	− 64.1
*p *values			Year < .001			Year < .001		
Walking	Metro	1602	6.86	8.84	28.7	56.58	53.53	− 5.4
	Non-metro	963	6.69	8.25	23.4	53.61	52.05	− 2.9
	All	2565	6.80	8.62	26.8	55.47	52.97	− 4.5
*p *values			Year < .001					
Yoga	Metro	186	2.30	2.83	23.3	42.45	27.34	− 35.6
	Non-metro	122	3.38	4.02	18.9	40.54	24.68	− 39.1
	All	308	2.72	3.30	21.2	41.69	26.31	− 36.9
*p *values			Year .007 Region < .001			Year < .001		
Other activities^7,8^	Metro	747	2.65	1.42	− 46.4	77.45	27.48	− 64.5
	Non-metro	418	3.38	2.13	− 36.9	88.09	33.70	− 61.7
	All	1165	2.91	1.68	− 42.4	81.27	29.71	− 63.4
All activities^8^	Metro	11,303	4.37	3.06	− 30.1	91.09	37.56	− 58.8
	Non-metro	5995	4.46	3.07	− 31.1	95.92	38.34	− 60.0
	All	17,298	4.40	3.06	− 30.4	92.76	37.83	− 59.2

^1^Of the 6140 survey respondents 5371 answered the questions on sport and physical activities. They reported 19,205 instances of participation (i.e. a person reporting participation in a particular activity) in a total of 88 sports and physical activities—an average of 3.6 different activities per person. The 18 activities which each contributed at least 1% of all instances of participation are listed in this table

^2^The metropolitan region comprises the Greater Capital City Statistical Area of each Australian state and territory (Australian Bureau of Statistics ref). The non-metropolitan region comprises the remainder of Australia. Regions are defined on the basis of residential postcode

^3^Some respondents did not answer the frequency and duration questions or the postcode question, and so the total number of responses is 17,298 rather than 19,205. A few gave partial responses, and so the sample sizes for the four variables in this table are not exactly equal. The indicative sample sizes shown in the table are for the frequency before Covid

^4^Question wording for frequency: “Before the Coronavirus (COVID-19) pandemic, in a usual two-week period during the season for each of these sports, how many times would you participate (including any training or practice)?”; “In the past two weeks, how many times have you participated in each of these sports (including any training or practice)?”

^5^Question wording for duration: “On average, how many minutes would each session last?”; “On average, how many minutes does each session last?”

^6^Only statistically significant *p *values (< .05) are shown in the table

^7^ ‘Other’ encompasses the 70 activities which each contributed less than 1% of all reported instances of participation

^8^No statistical significance tests were conducted for data aggregated across multiple activities because many respondents participated in more than one activity, violating the assumption of independent observations

**Table 3:** Changes in self-reported frequency and duration of 18 popular forms of sport and physical activity^1^ from before Covid to during Covid: adults (aged 18 +) by gender

Activity	Gender^2^	n^2,3^	Frequency^4^ (Sessions per two weeks)			Duration^5^ (Minutes per session)		
*p *values^6^			Mean before Covid	Mean during Covid	Change %	Mean before Covid	Mean during Covid	Change %
Australian football	Male	414	2.76	0.92	− 66.5	68.48	24.22	− 64.6
	Female	87	2.49	0.46	− 81.6	63.38	14.94	− 76.4
	All	502	2.71	0.84	− 68.9	67.60	22.57	− 66.6
*p *values			Year < .001			Year < .001		
Basketball	Male	104	2.54	1.08	− 57.7	48.13	16.93	− 64.8
	Female	76	2.16	0.91	− 58.0	44.41	9.34	− 79.0
	All	180	2.38	1.01	− 57.8	46.56	13.80	− 70.4
*p *values			Year < .001			Year < .001 Gender .047		
Bowls	Male	796	6.24	0.54	− 91.4	152.65	37.02	− 75.7
	Female	288	5.90	0.33	− 94.4	171.07	37.01	− 78.4
	All	1091	6.20	0.48	− 92.3	157.57	36.94	− 76.6
*p *values			Year < .001			Year < .001 Year x Gender .023		
Boxing	Male	148	2.27	1.07	− 53.0	38.38	14.11	− 63.2
	Female	49	1.64	3.09	87.8	31.70	18.24	− 42.5
	All	198	2.11	1.55	− 26.5	36.56	14.98	− 59.0
*p *values			Year x Gender .03			Year < .001		
Bush walking	Male	249	1.73	2.19	26.2	91.03	43.91	− 51.8
	Female	249	1.80	2.09	15.7	93.80	46.46	− 50.5
	All	500	1.76	2.13	20.9	92.48	45.13	− 51.2
*p *values			Year < .001					
Cricket	Male	436	3.23	0.62	− 80.9	137.81	34.34	− 75.1
	Female	44	1.95	0.64	− 67.1	88.05	6.59	− 92.5
	All	481	3.11	0.62	− 80.1	133.00	31.68	− 76.2
*p *values			Year < .001			Year < .001 Year x Gender .002		
Cycling	Male	597	3.45	3.88	12.4	62.10	58.43	− 5.9
	Female	313	3.02	3.48	15.2	50.28	51.70	2.8
	All	913	3.30	3.74	13.3	57.96	56.07	− 3.3
*p *values						Gender .015		
Fitness/Gym	Male	699	4.88	2.93	− 40.0	56.01	26.47	− 52.7
	Female	601	4.23	3.21	− 24.2	54.32	26.35	− 51.5
	All	1303	4.58	3.06	− 33.2	55.19	26.40	− 52.2
*p *values			Year < .001 Year x Gender .003			Year < .001		
Football/soccer	Male	608	4.70	1.43	− 69.6	90.34	26.54	− 70.6
	Female	243	4.34	1.26	− 71.1	85.56	23.92	− 72.0
	All	855	4.59	1.37	− 70.1	89.31	25.78	− 71.1
*p *values			Year < .001			Year < .001		
Golf	Male	1518	4.30	1.45	− 66.3	219.61	58.77	− 73.2
	Female	676	4.58	1.18	− 74.2	230.68	50.71	− 78.0
	All	2199	4.39	1.36	− 69.0	223.04	56.27	− 74.8
*p *values			Year < .001			Year < .001 Year x Gender .002		
Pilates	Male	66	2.55	1.44	− 43.3	52.73	27.50	− 47.8
	Female	232	2.22	1.95	− 12.3	46.63	24.02	− 48.5
	All	298	2.29	1.84	− 19.8	47.96	24.80	− 48.3
*p *values			Year < .001 Year x Gender .005			Year < .001 Gender .043		
Running/jogging	Male	492	3.32	4.35	31.1	35.09	31.49	− 10.3
	Female	293	3.04	4.71	54.6	35.29	34.43	− 2.4
	All	789	3.22	4.48	39.3	35.23	32.68	− 7.2
*p *values			Year < .001					
Swimming	Male	399	4.08	0.58	− 85.9	52.53	10.27	− 80.5
	Female	393	4.54	1.16	− 74.5	64.91	11.35	− 82.5
	All	796	4.32	0.87	− 79.9	58.63	10.80	− 81.6
*p *values			Year < .001 Gender .015			Year < .001 Gender .013 Year x Gender .006		
Tennis	Male	403	3.42	1.58	− 53.9	104.27	41.70	− 60.0
	Female	334	3.08	1.12	− 63.6	114.32	33.83	− 70.4
	All	740	3.27	1.37	− 58.1	108.75	38.42	− 64.7
*p *values			Year < .001			Year < .001 Year x Gender .002		
Walking	Male	1303	7.12	8.68	21.9	54.03	51.68	− 4.4
	Female	1045	6.61	8.86	34.1	59.97	56.38	− 6.0
	All	2353	6.89	8.76	27.1	56.67	53.78	− 5.1
*p *values			Year < .001			Gender .006		
Yoga	Male	74	3.09	3.23	4.4	34.47	21.34	− 38.1
	Female	227	2.60	3.35	28.7	44.56	28.28	− 36.5
	All	305	2.72	3.32	21.9	41.85	26.54	− 36.6
*p *values			Year < .001			Gender .001		
Other activities ^7,8^	Male	520	2.74	1.26	− 53.9	86.22	30.78	− 64.3
	Female	554	3.20	1.82	− 43.0	74.80	24.28	− 67.5
	All	1079	3.01	1.55	− 48.6	80.13	27.39	− 65.8
All activities^8^	Male	8826	4.44	2.80	− 36.9	103.10	39.98	− 61.2
	Female	5704	4.18	3.29	− 21.3	89.70	36.66	− 59.1
	Other^2^	31	5.35	2.10	− 60.8	67.90	16.83	− 75.2
	No response^2^	21	7.57	2.85	− 62.4	121.90	40.95	− 66.4
	All	14,582	4.34	2.99	− 31.2	97.81	38.65	− 60.5

^1^Of the 4862 survey respondents identified by age as adult 4724 answered the questions on sport and physical activities. They reported 16,117 instances of participation (i.e. a person reporting participation in a particular activity) in a total of 88 sports and physical activities—an average of 3.4 different activities per person. The 15 activities which each contributed at least 1% of all instances of participation are listed in this table

^2^Four gender response categories were provided: ‘Male’, ‘Female’, ‘Other’, and ‘Choose not to (abbreviated to ‘No response’ in the table). The ‘Other’ and ‘No response’ categories had counts, across all activities, of 31 and 21 respectively. The maximum count for an individual activity was 6. With such small sample sizes, the evidence base is small and the sampling variability is extremely large, and so for these categories the results for individual activities are not included in the table. For the aggregate results of all activities combined, results for all four gender categories are included

^3^Some respondents did not answer the frequency and duration questions, and so the total number of responses is 14,582 rather than 16,117. A few gave partial responses, and so the sample sizes for the four variables in this table are not exactly equal. The indicative sample sizes shown in the table are for the frequency before Covid

^4^Question wording for frequency: “Before the Coronavirus (COVID-19) pandemic, in a usual two-week period during the season for each of these sports, how many times would you participate (including any training or practice)?”; “In the past two weeks, how many times have you participated in each of these sports (including any training or practice)?”

^5^Question wording for duration: “On average, how many minutes would each session last?”; “On average, how many minutes does each session last?”

^6^Only statistically significant *p *values (< .05) are shown in the table

^7^ ‘Other’ encompasses the 73 activities which each contributed less than 1% of all reported instances of participation

^8^No statistical significance tests were conducted for data aggregated across multiple activities because many respondents participated in more than one activity, violating the assumption of independent observations

**Table 4:** Changes in self-reported frequency and duration of 18 popular forms of sport and physical activity^1^ from before Covid to during Covid: adults (aged 18 +) by region

Activity	Region^2^	n^3^	Frequency^4^ (sessions per 14 days)			Duration^5^ (minutes per session)		
*p *values^6^			Mean before Covid	Mean during Covid	Change %	Mean before Covid	Mean during Covid	Change %
Australian football	Metro	298	2.57	0.75	− 70.7	68.66	23.49	− 65.8
	Non-metro	203	2.93	0.97	− 66.8	66.43	21.36	− 67.8
	All	501	2.72	0.84	− 69.0	67.74	22.62	− 66.6
*p *values			Year < .001			Year < .001		
Basketball	Metro	123	2.39	1.07	− 55.0	44.96	15.61	− 65.3
	Non-metro	55	2.38	0.84	− 64.8	50.63	10.00	− 80.2
	All	178	2.38	1.00	− 58.1	46.74	13.96	− 70.1
*p *values			Year < .001			Year < .001 Year x Region .049		
Bowls	Metro	706	6.49	0.51	− 92.2	160.93	35.85	− 77.7
	Non-metro	382	5.67	0.43	− 92.5	151.80	39.31	− 74.1
	All	1088	6.20	0.48	− 92.3	157.75	37.05	− 76.5
*p *values			Year < .001			Year < .001		
Boxing	Metro	133	1.87	0.97	− 48.1	38.06	14.24	− 62.6
	Non-metro	63	2.58	2.76	6.9	32.58	15.53	− 52.3
	All	196	2.10	1.54	− 26.4	36.32	14.65	− 59.7
*p *values			–			Year < .001		
Bush walking	Metro	289	1.71	1.93	12.9	95.84	45.45	− 52.6
	Non-metro	211	1.84	2.40	30.6	87.95	44.70	− 49.2
	All	500	1.76	2.13	20.9	92.48	45.13	− 51.2
*p *values			–			Year < .001		
Cricket	Metro	330	3.05	0.54	− 82.5	133.85	32.77	− 75.5
	Non-metro	148	3.22	0.79	− 75.6	131.35	29.94	− 77.2
	All	478	3.10	0.61	− 80.3	133.09	31.90	− 76.0
*p *values			Year < .001			Year < .001		
Cycling	Metro	571	3.42	3.76	9.9	61.44	59.25	− 3.6
	Non-metro	340	3.11	3.71	19.0	52.38	50.88	− 2.9
	All	911	3.31	3.74	13.1	58.05	56.13	− 3.3
*p *values			–			–		
Fitness/Gym	Metro	910	4.58	3.08	− 32.7	55.36	27.11	− 51.0
	Non-metro	387	4.62	2.98	− 35.5	55.11	24.95	− 54.7
	All	1297	4.59	3.05	− 33.6	55.29	26.46	− 52.1
*p *values			Year < .001			Year < .001		
Football/soccer	Metro	666	4.58	1.36	− 70.4	87.16	26.34	− 69.8
	Non-metro	185	4.63	1.41	− 69.6	97.25	24.12	− 75.2
	All	851	4.59	1.37	− 70.2	89.35	25.85	− 71.1
*p *values			Year < .001			Year < .001 Year x Region .049		
Golf	Metro	1222	4.21	1.36	− 67.7	227.42	55.02	− 75.8
	Non-metro	973	4.63	1.37	− 70.4	217.69	57.91	− 73.4
	All	2195	4.39	1.36	− 69.0	223.11	56.30	− 74.8
*p *values			Year < .001			Year < .001		
Pilates	Metro	206	2.32	1.80	− 22.3	49.80	25.77	− 48.2
	Non-metro	92	2.23	1.92	− 13.9	43.85	22.50	− 48.7
	All	298	2.29	1.84	− 19.8	47.96	24.80	− 48.3
*p *values			Year .048			Year < .001		
Running/jogging	Metro	551	3.05	4.41	44.8	34.56	32.11	− 7.1
	Non-metro	235	3.65	4.66	27.5	36.96	34.15	− 7.6
	All	786	3.23	4.49	39.0	35.27	32.71	− 7.3
*p *values			Year < .001			Year .034		
Swimming	Metro	510	4.22	0.90	− 78.7	58.65	11.02	− 81.2
	Non-metro	281	4.53	0.82	− 81.8	58.76	10.48	− 82.2
	All	791	4.33	0.87	− 79.9	58.69	10.83	− 81.5
*p *values			Year < .001			Year < .001		
Tennis	Metro	526	3.41	1.31	− 61.5	110.63	39.92	− 63.9
	Non-metro	213	2.91	1.50	− 48.3	104.09	34.76	− 66.6
	All	739	3.27	1.37	− 58.1	108.74	38.48	− 64.6
*p *values			Year < .001			Year < .001		
Walking	Metro	1454	6.94	8.96	29.1	57.93	54.45	− 6.0
	Non-metro	893	6.84	8.45	23.5	54.78	52.88	− 3.5
	All	2347	6.91	8.77	27.0	56.73	53.85	− 5.1
*p *values			Year < .001			–		
Yoga	Metro	184	2.31	2.86	23.9	42.58	27.66	− 35.1
	Non-metro	121	3.36	4.02	19.7	40.75	24.76	− 39.2
	All	305	2.72	3.32	21.9	41.85	26.54	− 36.6
*p *values			Year.006 Region .001			Year < .001		
Other activities^7,8^	Metro	672	2.91	1.43	− 50.9	78.29	26.66	− 66.0
	Non-metro	405	3.18	1.74	− 45.3	83.36	28.61	− 65.7
	All	1077	3.01	1.55	− 48.7	80.19	27.38	− 65.9
All activities^8^	Metro	9351	4.31	2.95	− 31.4	96.34	38.18	− 60.4
	Non-metro	5187	4.41	3.05	− 30.9	100.74	39.70	− 60.6
	All	14,538	4.35	2.99	− 31.2	97.91	38.72	− 60.5

^1^Of the 4862 survey respondents identified by age as adult 4724 answered the questions on sport and physical activities. They reported 16,117 instances of participation (i.e. a person reporting participation in a particular activity) in a total of 88 sports and physical activities—an average of 3.4 different activities per person. The 15 activities which each contributed at least 1% of all instances of participation are listed in this table

^2^The metropolitan region comprises the Greater Capital City Statistical Area of each Australian state and territory (Australian Bureau of Statistics ref). The non-metropolitan region comprises the remainder of Australia. Regions are defined on the basis of residential postcode

^3^Some respondents did not answer the frequency and duration questions, and so the total number of responses is 14,538 rather than 16,117. A few gave partial responses, and so the sample sizes for the four variables in this table are not exactly equal. The indicative sample sizes shown in the table are for the frequency before Covid

^4^Question wording for frequency: “Before the Coronavirus (COVID-19) pandemic, in a usual 2-week period during the season for each of these sports, how many times would you participate (including any training or practice)?”; “In the past two weeks, how many times have you participated in each of these sports (including any training or practice)?”

^5^Question wording for duration: “On average, how many minutes would each session last?”; “On average, how many minutes does each session last?”

^6^Only statistically significant *p *values (< .05) are shown in the table

^7^ ‘Other’ encompasses the 73 activities which each contributed less than 1% of all reported instances of participation

^8^No statistical significance tests were conducted for data aggregated across multiple activities because many respondents participated in more than one activity, violating the assumption of independent observations

**Table 5:** Changes in self-reported frequency and duration of 18 popular forms of sport and physical activity^1^ from before Covid to during Covid: adolescents (aged 13–17) by gender

Activity	Gender^2^	n^3^	Frequency^4^ (sessions per 14 days)			Duration^5^ (minutes per session)		
*p *values^6^			Mean before Covid	Mean during Covid	Change %	Mean before Covid	Mean during Covid	Change %
Athletics	Male	48	1.67	1.20	− 28.3	40.89	18.33	− 55.2
	Female	28	6.29	2.50	− 60.2	51.43	33.93	− 34.0
	All	76	3.37	1.69	− 49.9	44.83	24.57	− 45.2
*p *values						Year < .001		
Australian football	Male	51	2.73	1.69	− 37.9	54.80	25.31	− 53.8
	Female	43	2.79	0.91	− 67.5	69.74	14.30	− 79.5
	All	94	2.76	1.33	− 51.9	61.78	20.11	− 67.5
*p *values			Year < .001			Year < .001 Year x Gender .042		
Basketball	Male	111	3.41	3.19	− 6.5	46.62	27.32	− 41.4
	Female	35	2.54	3.09	21.3	43.09	14.85	− 65.5
	All	146	3.21	3.17	− 1.2	45.78	24.36	− 46.8
*p *values						Year < .001		
Cricket	Male	67	2.69	0.33	− 87.6	69.85	16.39	− 76.5
	Female	20	3.65	0.55	− 84.9	61.00	18.95	− 68.9
	All	87	2.91	0.38	− 86.8	67.79	17.00	− 74.9
*p *values			Year < .001			Year < .001		
Cycling	Male	101	3.80	5.47	43.9	55.81	53.65	− 3.9
	Female	66	1.35	3.33	147.2	33.15	46.36	39.8
	All	169	2.86	4.64	62.5	46.44	50.55	8.8
*p *values			Year < .001 Gender < .001			Year .038 Gender .033		
Dancing	Male	2	2.00	2.00	0.0	40.00	30.00	− 25.0
	Female	25	4.88	5.04	3.3	51.04	41.00	− 19.7
	All	27	4.67	4.92	5.4	50.60	40.56	− 19.8
*p *values			–			–		
Fitness/Gym	Male	131	5.10	5.83	14.3	50.82	42.30	− 16.8
	Female	106	3.97	5.73	44.4	53.19	33.43	− 37.1
	All	237	4.59	5.79	25.9	51.88	38.25	− 26.3
*p *values			Year .012			Year < .001		
Football/soccer	Male	365	6.93	4.35	− 37.3	89.81	45.87	− 48.9
	Female	129	4.84	2.63	− 45.8	85.78	35.25	− 58.9
	All	497	6.40	3.89	− 39.2	88.93	43.10	− 51.5
*p *values			Year < .001 Gender .001			Year < .001		
Golf	Male	28	2.75	2.50	− 9.1	121.85	31.85	− 73.9
	Female	4	1.75	0.00	− 100.0	30.00	0.00	− 100.0
	All	32	2.63	2.17	− 17.5	110.00	27.74	− 74.8
*p *values						Year .021 Gender .028		
Netball	Male	4	1.75	0.75	− 57.1	26.25	12.50	− 52.4
	Female	62	3.02	0.74	− 75.4	65.68	18.39	− 72.0
	All	68	2.97	0.72	− 75.7	64.37	17.46	− 72.9
*p *values			Year .002					
Running/jogging	Male	144	4.69	6.03	28.4	34.76	35.76	2.9
	Female	110	3.30	4.43	34.1	28.89	32.48	12.4
	All	254	4.09	5.33	30.3	32.26	34.33	6.4
*p *values			Year .014					
Skating/scootering	Male	19	2.95	1.94	− 34.1	43.42	29.71	− 31.6
	Female	17	3.82	3.53	− 7.7	37.35	35.59	− 4.7
	All	36	3.36	2.74	− 18.6	40.56	32.65	− 19.5
*p *values			–			–		
Surf Lifesaving	Male	14	1.50	0.46	− 69.2	137.86	19.09	− 86.2
	Female	18	3.06	0.39	− 87.3	74.44	16.47	− 77.9
	All	32	2.38	0.42	− 82.3	102.19	17.50	− 82.9
*p *values			Year < .001 Time x Gender .015			Year < .001		
Swimming	Male	120	5.71	1.43	− 74.9	77.96	22.20	− 71.5
	Female	172	9.42	1.78	− 81.1	106.92	18.85	− 82.4
	All	294	7.86	1.63	− 79.3	95.04	20.02	− 78.9
*p *values			Year < .001 Gender < .001 Time x Gender < .001			Year < .001 Gender .001 Time x Gender < .001		
Tennis	Male	81	3.56	0.73	− 79.4	64.29	26.70	− 58.5
	Female	32	5.13	1.94	− 62.2	77.90	29.80	− 61.7
	All	113	4.00	1.08	− 72.9	68.19	27.64	− 59.5
*p *values			Year < .001 Gender .002			Year < .001		
Volleyball	Male	12	1.58	0.00	− 100.0	29.17	24.09	− 17.4
	Female	12	1.17	0.00	− 100.0	48.75	3.33	− 93.2
	All	26	1.58	0.00	− 100.0	43.65	12.20	− 72.1
*p *values			Year < .001 Gender .002			Year .001 Time x Gender .001		
Walking	Male	87	5.41	7.60	40.3	43.31	38.48	− 11.2
	Female	104	5.68	6.20	9.1	38.59	46.07	19.4
	All	193	5.60	6.88	22.9	40.58	42.92	5.8
*p *values			Year.015			Year .035		
Other activities^7,8^	Male	75	3.13	1.93	− 38.4	65.76	32.31	− 50.9
	Female	82	3.14	2.72	− 13.5	84.64	37.89	− 55.2
	All	160	3.14	2.32	− 26.0	76.55	34.79	− 54.6
All activities^8^	Male	1460	4.73	3.75	− 20.6	64.90	35.79	− 44.9
	Female	1065	4.73	3.15	− 33.4	65.06	30.43	− 53.2
	No response^2^	16	5.25	3.06	− 41.7	84.69	21.88	− 74.2
	All	2541	4.73	3.49	− 26.2	65.10	33.40	− 48.7

^1^Of the 584 survey respondents identified by age as adolescent, 580 answered the questions on sport and physical activities. They reported 2822 instances of participation (i.e. a person reporting participation in a particular activity) in a total of 88 sports and physical activities—an average of 4.9 different activities per person. The 17 activities which each contributed at least 1% of all instances of participation are listed in this table

^2^Four gender response categories were provided: ‘Male’, ‘Female’, ‘Other’, and ‘Choose not to respond’ (abbreviated to ‘No response’ in the table). The ‘Other’ and ‘No response’ categories had counts, across all activities, of 0 and 16 respectively. The maximum count for an individual activity was 3. With such small sample sizes, the evidence base is small and the sampling variability is extremely large, and so for this category the results for individual activities are not included in the table. For the aggregate results of all activities combined, results for all four gender categories are included

^3^Some respondents did not answer the frequency and duration questions, and so the total number of responses is 2541 rather than 2822. A few gave partial responses, and so the sample sizes for the four variables in this table are not exactly equal. The indicative sample sizes shown in the table are for the frequency before Covid

^4^Question wording for frequency: “Before the Coronavirus (COVID-19) pandemic, in a usual 2-week period during the season for each of these sports, how many times would you participate (including any training or practice)?”; “In the past two weeks, how many times have you participated in each of these sports (including any training or practice)?”

^5^Question wording for duration: “On average, how many minutes would each session last?”; “On average, how many minutes does each session last?”

^6^Only statistically significant *p *values (< .05) are shown in the table

^7^ ‘Other’ encompasses the 71 activities which each contributed less than 1% of all reported instances of participation

^8^No statistical significance tests were conducted for data aggregated across multiple activities because many respondents participated in more than one activity, violating the assumption of independent observations

**Table 6:** Changes in self-reported frequency and duration of 18 popular forms of sport and physical activity^1^ from before Covid to during Covid: adolescents (aged 13–17) by region

Activity	Region^2^	n^3^	Frequency^4^ (sessions per 14 days)			Duration^5^ (minutes per session)		
*p *values^6^			Mean before Covid	Mean during Covid	Change %	Mean before Covid	Mean during Covid	Change %
Athletics	Metro	59	2.15	1.68	− 21.8	45.38	28.06	− 38.2
	Non-metro	17	7.59	1.71	− 77.5	42.94	12.81	− 70.2
	All	76	3.37	1.69	− 49.9	44.83	24.57	− 45.2
*p *values			Year .018			Year < .001		
Australian football	Metro	61	2.56	1.71	− 33.1	58.05	24.48	− 57.8
	Non-metro	33	3.12	0.64	− 79.6	68.45	12.42	− 81.9
	All	94	2.76	1.33	− 51.9	61.78	20.11	− 67.5
*p *values			Year < .001 Year x Region .023			Year < .001		
Basketball	Metro	109	3.24	3.58	10.7	45.21	25.35	− 43.9
	Non-metro	37	3.11	2.00	− 35.7	47.50	21.43	− 54.9
	All	146	3.21	3.17	− 1.2	45.78	24.36	− 46.8
*p *values			–			Year < .001		
Cricket	Metro	58	2.88	0.54	− 81.1	71.58	21.23	− 70.3
	Non-metro	29	2.97	0.07	− 97.7	60.34	8.70	− 85.6
	All	87	2.91	0.38	− 86.8	67.79	17.00	− 74.9
*p *values			Year < .001			Year < .001		
Cycling	Metro	116	2.77	4.52	63.3	50.57	53.94	6.7
	Non-metro	53	3.06	4.92	61.1	37.31	43.04	15.4
	All	169	2.86	4.64	62.5	46.44	50.55	8.8
*p *values			Year < .001			–		
Dancing	Metro	18	4.17	5.00	20.0	46.25	41.50	− 10.3
	Non-metro	9	5.67	4.78	− 15.7	58.33	38.89	− 33.3
	All	27	4.67	4.92	5.4	50.60	40.56	− 19.8
*p *values			–			–		
Fitness/gym	Metro	170	4.50	5.98	32.9	53.53	40.37	− 24.6
	Non-metro	67	4.84	5.30	9.6	47.79	32.84	− 31.3
	All	237	4.59	5.79	25.9	51.88	38.25	− 26.3
*p *values			–			Year < .001		
Football/soccer	Metro	373	6.68	4.08	− 38.8	88.39	45.92	− 48.0
	Non-metro	124	5.57	3.33	− 40.2	90.52	34.59	− 61.8
	All	497	6.40	3.89	− 39.2	88.93	43.10	− 51.5
*p *values			Year < .001			Year < .001 Year x Region .031		
Golf	Metro	25	2.52	2.54	0.9	110.83	29.17	− 73.7
	Non-metro	7	3.00	0.67	− 77.8	107.14	22.86	− 78.7
	All	32	2.63	2.17	− 17.5	110.00	27.74	− 74.8
*p *values			–			Year < .001		
Netball	Metro	34	2.65	0.41	− 84.4	64.47	7.73	− 88.0
	Non-metro	34	3.29	1.03	− 68.8	64.26	27.50	− 57.2
	All	68	2.97	0.72	− 75.7	64.37	17.46	− 72.9
*p *values			Year < .001			Year < .001		
Running/jogging	Metro	180	3.94	5.60	41.9	35.09	36.21	3.2
	Non-metro	74	4.45	4.68	5.3	25.44	29.79	17.1
	All	254	4.09	5.33	30.3	32.26	34.33	6.4
*p *values			–			–		
Skating/scootering	Metro	24	3.79	3.36	− 11.3	39.17	38.18	− 2.5
	Non-metro	12	2.50	1.58	− 36.7	43.33	22.50	− 48.1
	All	36	3.36	2.74	− 18.6	40.56	32.65	− 19.5
*p *values			–			–		
Surf lifesaving	Metro	26	2.77	0.48	− 82.7	86.54	13.48	− 84.4
	Non-metro	6	0.67	0.17	− 75.0	170.00	36.00	− 78.8
	All	32	2.38	0.42	− 82.3	102.19	17.50	− 82.9
*p *values			Year .011			Year .001		
Swimming	Metro	204	7.69	1.43	− 81.5	96.61	19.77	− 79.5
	Non-metro	90	8.22	2.08	− 74.7	91.49	20.60	− 77.5
	All	294	7.86	1.63	− 79.3	95.04	20.02	− 78.9
*p *values			Year < .001			Year < .001		
Tennis	Metro	76	4.18	0.68	− 83.7	65.21	27.82	− 57.3
	Non-metro	37	3.62	1.84	− 49.3	73.92	27.27	− 63.1
	All	113	4.00	1.08	− 72.9	68.19	27.64	− 59.5
*p *values			Year < .001			Year < .001		
Volleyball	Metro	19	1.53	0.00	− 100.0	34.47	16.94	− 50.8
	Non-metro	7	1.71	0.00	− 100.0	68.57	0.00	− 100.0
	All	26	1.58	0.00	− 100.0	43.65	12.20	− 72.1
*p *values			Year < .001			Year < .001 Year x Region .005		
Walking	Metro	128	6.15	7.54	22.6	42.58	43.45	2.0
	Non-metro	65	4.51	5.62	24.6	36.69	41.92	14.3
	All	193	5.60	6.88	22.9	40.58	42.92	5.8
*p *values			Year .035			Year .028 Region .036		
Other activities^6,7^	Metro	98	2.18	1.45	− 33.7	71.74	27.67	− 61.4
	Non-metro	62	4.65	3.70	− 20.4	84.18	46.67	− 44.6
	All	160	3.14	2.32	− 26.0	76.55	34.79	− 54.6
All activities^7^	Metro	1778	4.72	3.61	− 23.5	65.54	34.79	− 46.9
	Non-metro	763	4.75	3.21	− 32.4	64.06	30.16	− 52.9
	All	2541	4.73	3.49	− 26.2	65.10	33.40	− 48.7

^1^Of the 584 survey respondents identified by age as adolescent, 580 answered the questions on sport and physical activities. They reported 2822 instances of participation (i.e. a person reporting participation in a particular activity) in a total of 88 sports and physical activities—an average of 4.9 different activities per person. The 17 activities which each contributed at least 1% of all instances of participation are listed in this table

^2^The metropolitan region comprises the Greater Capital City Statistical Area of each Australian state and territory (Australian Bureau of Statistics ref). The non-metropolitan region comprises the remainder of Australia. Regions are defined on the basis of residential postcode

^3^Some respondents did not answer the frequency and duration questions, and so the total number of responses is 2541 rather than 2822. A few gave partial responses, and so the sample sizes for the four variables in this table are not exactly equal. The indicative sample sizes shown in the table are for the frequency before Covid

^4^Question wording for frequency: “Before the Coronavirus (COVID-19) pandemic, in a usual 2-week period during the season for each of these sports, how many times would you participate (including any training or practice)?”; “In the past two weeks, how many times have you participated in each of these sports (including any training or practice)?”

^5^Question wording for duration: “On average, how many minutes would each session last?”; “On average, how many minutes does each session last?”

^6^‘Other’ encompasses the 71 activities which each contributed less than 1% of all reported instances of participation

^7^No statistical significance tests were conducted for data aggregated across multiple activities because many respondents participated in more than one activity, violating the assumption of independent observations

## Results

Of 6140 survey respondents, 5371 answered the questions on sport and physical activities, providing the basis for Tables 1 and 2. The supplementary tables are based on the 4724 identified as adults (Tables 3, 4) and 560 as adolescents (Tables 5, 6). Collectively, respondents reported 19,205 instances of participation (i.e., a person reporting that they participated in a particular activity), an average of 3.6 different activities per person, in a total of 88 sports and physical activities. In each pair of tables, results are shown for each activity that contributed at least 1% of all reported instances of participation by the particular group (18 for all respondents, 15 for adults and 17 for adolescents), with the remainder of the reported activities aggregated as ‘Other’.

### Gender differences in sport and physical activity participation

Of all sports and physical activities, before COVID-19, the participation frequency of men and boys (mean of 4.5 sessions in past two weeks) was slightly higher than that of women and girls (mean 4.3), however during COVID-19, the participation frequency of men and boys was lower (mean 2.9) than that of women and girls (mean 3.3) (Table 1). The percentage decreases in mean frequency of participation were 35% and 24% respectively, and much greater (61%) for people who did not identify as man or woman.

Similarly, the pre-COVID-19 mean duration of activity sessions (97 min for men and boys, 86 min for women and girls) declined slightly more for men and boys (60%) than women and girls (59%) (Table 1). Further, those who did not identify as man/woman or boy/girl had a greater decline in duration (75%).

For adults, overall men’s mean participation frequency and duration declined more (37%, 61%) than women’s (21%, 59%) (Table 3). However, this pattern was reversed for adolescents, with mean participation frequency and duration of girls declining more (33%, 53%) than for boys (21%, 45%) (Table 5).

### Differences between specific sport and physical activities

The majority of the 18 most popular sports and physical activities across all age groups had an overall decline in mean participation frequency during COVID-19. The sports and physical activities with the greatest overall decline were bowls (92%), cricket (81%), swimming (80%) and netball (77%) (Table 1). The decrease in bowls and cricket is likely to be related to a season effect, with these being summer sports. The sports and physical activities with the largest decrease in mean duration were swimming (80%), netball (77%), bowls (77%) and cricket (76%). Except for swimming, these are all team sports. Within these sports and activities, all genders had relatively similar patterns of decline in mean frequency, and also reflected the gendered nature of some sports such as Australian football, and cricket which are male-dominated (Table 1).

In contrast, five sports and physical activities demonstrated increases in mean frequency of participation during COVID-19; these were running/jogging (36%), walking (27%), yoga (21%), bushwalking (20%) and cycling (20%) (Table 1). For all of these activities except bushwalking, women and girls had a higher increase in participation than men and boys. Further, for boxing, participation of women and girls increased by 79%, while participation of men and boys in boxing decreased. The only increases in duration were for women and girls in cycling and running/jogging.

### Regional differences

Pre-COVID-19, overall participation in the 18 most popular sports and physical activities was slightly higher for non-metropolitan residents, for both frequency and duration (mean 4.5 sessions and 96 min) compared to metropolitan residents (mean 4.4 sessions and 91 min) (Table 2). Participation decreased slightly more for non-metropolitan residents, both in frequency and duration (31%; 60%) than for metropolitan residents (30%; 59%).

When examining changes in frequency of participation across the diverse sports and physical activities, for most activities, participation decreased in both metropolitan and non-metropolitan areas. Basketball, athletics and tennis had the greatest regional differences, with non-metropolitan areas having a much greater participation decline in basketball and athletics, and metropolitan areas having a much greater decline in tennis (Table 2).

Participation during COVID-19 increased in both regions for running/jogging, walking, bushwalking, cycling and yoga. Participation in boxing increased in non-metropolitan areas only (Table 2). This was consistent for the overall sample and for adults aged 18 years and over (Table 4).

Regional comparison of increases in participation frequency shows that non-metropolitan areas had larger increases in the frequency of participation in bushwalking, boxing, and cycling, while metropolitan areas had larger increases in the frequency of participation in running/jogging, walking and yoga (Table 2). With the exception of boxing, this pattern of regional differences was also apparent for adult respondents (Table 4).

Adolescents living in both metropolitan and non-metropolitan regions increased their frequency of participation in cycling, fitness/gym, running/jogging and walking, with the largest increase by far in cycling. Adolescents in metropolitan regions also increased their frequency of participation in basketball, dancing and golf (Table 6).

Adolescents increased their duration of participation for more activities than adults, including cycling, running/jogging and walking. Non-metropolitan regions had greater increases in duration of participation than metropolitan regions in all of these activities (Table 6).

## Discussion

This study investigated the changes in the frequency and duration of participation in different sports and activities by Australian adults and adolescents due to COVID-19 restrictions. Globally, many studies have investigated changes in total physical activity [11, 23], however there has been little research into changes in specific activities. This level of detail is important from a sports management and public health perspective because both sports organizations and government need specific information on the relative impact of COVID-19 on different organizations and their participants, to target investment and strategic developments throughout the COVID-19 recovery phase.

Two of our findings stand out—one unsurprising and one unexpected. First, we found that the greatest decline in participation—across the board—was in club team sports. It stands to reason that under pandemic conditions when social distance is required, team sports, particularly close-contact team sports, will suffer. This is further exacerbated by the fact that many public facilities where team sport is played had to close. Conversely, as to be expected, there was increased participation in individual activities, in activities in which social distancing was easier to achieve and in activities that did not depend on either limited-access facilities or on organized (timely access) activities.

Second, we found that in general the frequency of participation of men showed a steeper decline than that of women. This is contrary to many other studies that have found that women and girls participation and health declined more under COVID-19 than that of boys and men [14, 24, 25]. This is reportedly due to the additional impact on women of the care burden of children, home schooling, and looking after sick family members, and of women being more impacted by job losses [14, 24, 25]. However, there is also other evidence that individuals who were active within sports clubs were less likely to exercise during COVID-19 restrictions [26]. The men may have had a greater decline as they are more likely to participate in organized sport, which was most impacted by COVID-19 restrictions [27]. Whereas women pre-pandemic are more likely to participate in other activities like walking and yoga and Pilates [28]. Therefore, it seems that, at least in an Australian context, where participation rates of men in organized sport are generally significantly higher than women, under conditions of a pandemic it might well be the case that women are more versatile and flexible in regard to transitioning between various means of engaging in sport and/or physical activity. This is consistent with other literature that reports that in general there was a shift from community activities to home-based activities [11]. Further, there is evidence that women enjoyed physical activity during confinement more than men and that women used social media and other resources to drive their physical activity [29]. These authors also reported that in pre-COVID times, men were more likely to be active outdoors and were less able to adapt to indoor activities than women [29]. Having said that, even with an influx of online activities available, it is consistently reported that individuals do not maintain their activity levels during home isolation [23, 30, 31]. Further, pre-COVID-19, women were more likely to be engaged in indoor activities such as yoga and Pilates than men [32], and may have been able to adapt with the use of online media to maintain these activities whilst at home. COVID-19 has impacted male and female participation in sport and physical activity quite differently, which is related to social aspects of participation and preferences for outdoor versus indoor activities [29].

We note, in that regard, that an obsessive focus (through government policy) on maximizing sport participation in club environments might not be the most effective way of stimulating population physical activity levels. Rather, the need to open up a broader range of opportunities beyond club settings may well be a learning drawn from the enforced closure of club sport environments. We know that whilst many people play club sport, many more, especially adults, play in settings other than clubs [8]. We have previously argued that sport needs to consider new strategies and participation options to attract and retain players [8, 33] and the changes to participation brought about by the COVID-19 pandemic reinforce this message. At a population level, competitive sport alone is not going to solve the physical activity crisis as evidenced by population participation trends [33].

In this current study, no substantial differences were observed between metropolitan and non-metropolitan areas. Pre-COVID-19, non-metropolitan residents were generally active at slightly higher levels, and the declines in participation during COVID-19 were quite similar for both metropolitan and non-metropolitan residents. Given there were greater COVID-19 lockdown restrictions in metropolitan cities in Australia, it was anticipated that rural and regional residents would have maintained higher participation rates than those in metropolitan cities. However, there are also potentially countervailing factors. We know that individuals living in areas with lower socio-economic status were less likely to be physical active, both before and during COVID-19 [24], and regional and rural areas in Australia are generally lower in socio-economic status than metropolitan areas, and also tend to have poorer health profiles and poorer access to health care [34]. Such differences may be amplified during a pandemic.

As expected, there were also age-related differences in participation. These are related to preferred activities, patterns of behavior and access to equipment, which differ across the lifespan, with children and adolescents being more likely to participate in sport, and adults in other non-sport related physical activity [4, 8, 33]. Not surprisingly, in this study adults increased frequency in running/jogging, walking, yoga and cycling. However, while adolescents also increased their frequency in cycling, those in metropolitan areas also increased their frequency in basketball, dancing and golf.

### Limitations

This study was based on data from a convenience sample, predominantly of Australian sports participants recruited with the assistance of NSOs and SSOs of four sports, in May and June 2020. The primary sample was supplemented by recruitment through social media, which resulted in an additional smaller sample of participants in only informal sport or other physical activity settings. Consequently, the sample is subject to both known and unknown sources of bias, and caution must be exercised in generalizing the results. Even within the primary club sport sample, the geographical coverage was uneven, depending on the strength of the relationships between the research team and the SSOs in the various states, and the capacities and priorities of different SSOs in the context of the unfolding COVID-19 situation. Nevertheless, on the other side of the ledger, the sample obtained was extremely large, and because respondents provided information about the multiple sports and other physical activities that they engaged in, there was comprehensive representation of the sporting codes and other types of recreational physical activity that are available in Australia.

## Conclusions

Even before COVID-19, societal patterns of participation in sport and physical activity were changing from organized, competitive, and structured activities to activities with more time and place related flexibility, with more social options and more informal participation [33]. It is important that providers of sport and physical activity opportunities recognize changing consumer behavior and preferences in developing strategies to promote participation in sport and physical activity [33]. Overlaying this are the complexities with returning to play sport which includes general hygiene measures, returning to play after being infected, as well as significant changes to play and training to accommodate social distancing. This includes a ‘get in, train, get out’ mentality [35]. In this Australian study, the greatest decline in participation during COVID-19 was in team sport. Clearly, due to factors including seasonality, the contact nature of participation, and general age of participants, sports were impacted in different ways and to different degrees, and pandemic recovery plans needs to be prioritized to those sports impacted most heavily. Further, the decline in participation was greater for men than for women, largely because men were more likely to play club-based competitive sport which was banned during COVID-19 restrictions. It will be interesting to observe how sport will respond to getting these men back in the game, and women back from home-based yoga and Pilates. COVID-19 may possibly be the ‘perfect storm’ for sports and physical activity stakeholder organizations to reassess and provide a wider range of programs to suit a changing and diverse consumer demand.

## Data Availability

The datasets generated and/or analysed during the current study are not publicly available due to ethical arrangements, but are available from the corresponding author on reasonable request.
